# On the whereabouts of SARS-CoV-2 in the human body: A systematic review

**DOI:** 10.1371/journal.ppat.1009037

**Published:** 2020-10-30

**Authors:** Wim Trypsteen, Jolien Van Cleemput, Willem van Snippenberg, Sarah Gerlo, Linos Vandekerckhove

**Affiliations:** HIV Cure Research Center, Department of Internal Medicine and Pediatrics, Ghent University & Ghent University Hospital, Ghent, Belgium; Yale University School of Medicine, UNITED STATES

## Abstract

Since SARS-CoV-2 appeared in the human population, the scientific community has scrambled to gather as much information as possible to find good strategies for the containment and treatment of this pandemic virus. Here, we performed a systematic review of the current (pre)published SARS-CoV-2 literature with a focus on the evidence concerning SARS-CoV-2 distribution in human tissues and viral shedding in body fluids. In addition, this evidence is aligned with published ACE2 entry-receptor (single cell) expression data across the human body to construct a viral distribution and ACE2 receptor body map. We highlight the broad organotropism of SARS-CoV-2, as many studies identified viral components (RNA, proteins) in multiple organs, including the pharynx, trachea, lungs, blood, heart, vessels, intestines, brain, male genitals and kidneys. This also implicates the presence of viral components in various body fluids such as mucus, saliva, urine, cerebrospinal fluid, semen and breast milk. The main SARS-CoV-2 entry receptor, ACE2, is expressed at different levels in multiple tissues throughout the human body, but its expression levels do not always correspond with SARS-CoV-2 detection, indicating that there is a complex interplay between virus and host. Together, these data shed new light on the current view of SARS-CoV-2 pathogenesis and lay the foundation for better diagnosis and treatment of COVID-19 patients.

## Author summary

Since the beginning of 2020, SARS-CoV-2 quickly spread throughout the human population and caused a pandemic with devastating consequences at a global scale. The scientific community is challenged to find good strategies for the containment and treatment of this virus. In this context, an important step is charting the viral presence in the human body to improve diagnostics, prevention or treatment.

Here, we bring together the current scientific knowledge on SARS-CoV-2 detection in the human body and body fluids. We observe that SARS-CoV-2 impacts the human body well beyond the lungs and shows a complex interplay with the human host that is not always correlated with its entry receptor (ACE2) expression levels. Many studies identified viral components (RNA, proteins) of SARS-CoV-2 in multiple organs (pharynx, trachea, lungs, blood, heart, vessels, intestines, brain, male genitals and kidneys) and body fluids (mucus, saliva, urine, cerebrospinal fluid, semen and breast milk). However, besides the lungs, researchers were only able to detect infectious virus in stool and urine in a limited set of SARS-CoV-2 patients.

By combining these studies, our study provides an eagle’s view on the current status of SARS-CoV-2 pathogenesis and lays the foundation for better diagnosis and treatment of COVID-19 patients.

## Introduction

Coronavirus disease (COVID-19) is considered one of the largest fast expanding pandemics since the 1918 Spanish flu with serious consequences for global health and economy. As of July 1^st^ 2020, the coronavirus causing COVID-19, severe acute respiratory syndrome coronavirus 2 (SARS-CoV-2), has infected more than 10 million people and caused over half a million deaths (WHO Coronavirus Disease Dashboard, https://covid19.who.int). These numbers overshadow the impact of the related SARS coronavirus (SARS-CoV), which caused about 8,000 infections and 800 deaths [1]. As for SARS-CoV, SARS-CoV-2 is believed to be a derivative of an animal coronavirus that adopted the ability of human-to-human transmission [2]. However, in contrast to SARS-CoV, the more contagious SARS-CoV-2 rapidly spread around the world after it took off from a few human pilot infections in Wuhan, China.

SARS-CoV-2 spreads from one person to another through direct contact or over short distances in the air, either impacted in aerosol droplets or carried on fomites. Upon inhalation, SARS-CoV-2 enters host respiratory cells via interaction with its entry receptor, angiotensin-converting enzyme 2 (ACE2) and an activating receptor, a protease such as TMPRSS2 or cathepsin [3]. Viral replication in these cells elicits direct adverse effects on cells, but also induces local immune cells to quickly and abundantly secrete cytokines and chemokines. In turn, an excessive amount of immune cells are attracted to the site of infection causing a cascade of inflammatory reactions with detrimental effects on the lungs. The current view on SARS-CoV-2 pathogenesis focuses on these respiratory pathologies, causing symptoms such as coughing, fever, general malaise, dyspnea and respiratory distress, that might eventually lead to death [4]. However, increasing evidence shows that SARS-CoV-2 is not always confined to the respiratory tract, but may also spread to other organs. Indeed, the majority of COVID-19 patients show various other symptoms besides respiratory disorders including neurological, cardiovascular, intestinal and kidney malfunctions [5–11]. The pathophysiological mechanisms behind the latter symptoms are not yet fully understood. Researchers proposed viral-induced endothelial disturbances (e.g. thrombosis, hemorrhages and edema) and defective immune responses (e.g. cytokine storm or auto-immune reactions) as underlying reasons for multi-organ failure [5, 6]. In addition, direct viral replication in these organs may also account for multi-organ pathologies. In this context, the host cell entry receptor of SARS-CoV-2, ACE2, has been detected in cells from multiple tissues, including the respiratory tree, cornea, esophagus, ileum, colon, gallbladder and common bile duct tissues [12–14]. This raises the possibility for SARS-CoV-2 to engage with its receptor at multiple organ sites upon viremia, swallowing or axonal transport and cause organ-specific malfunctions. This replication in specific organs would enable the virus to be shed in multiple body fluids and would augment the chances for viral transmission to new hosts.

In order to get better insight into the different organs involved in SARS-CoV-2 infection, we performed a systematic narrative review on (pre-)published literature to determine which human organs and cell types are targeted by SARS-CoV-2. In addition, we sought to correlate the presence of SARS-CoV-2 with organ-specific expression of ACE2, the main entry receptor of SARS-CoV-2.

## Methods

### Systematic literature screening

**Fig 1:**
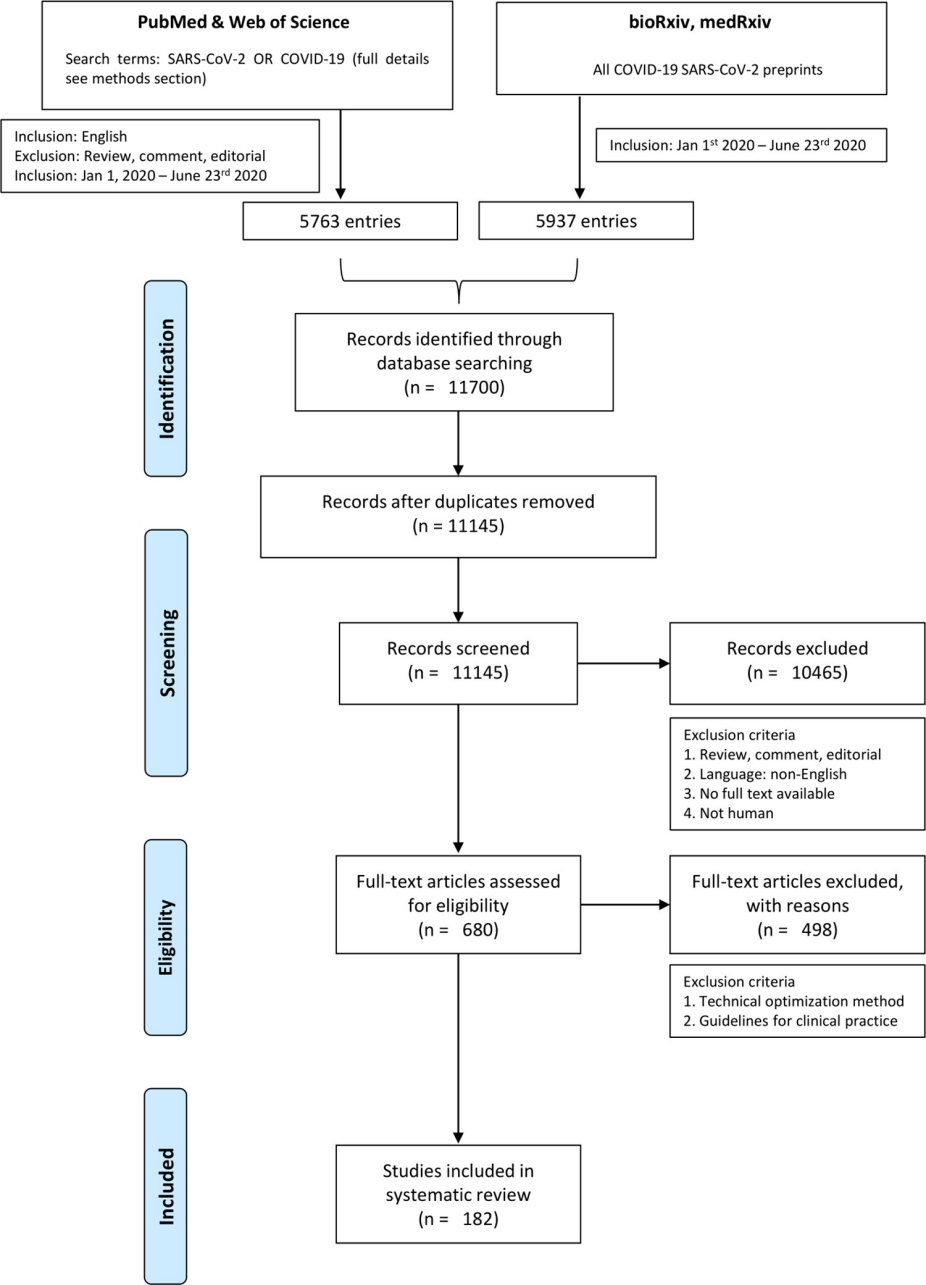
Overview of the systematic literature screening pipeline. Pubmed, Web of Science, BioRxiv and MedRxiv were used as database sources for the time period January 1^st^—June 23^rd^ 2020 and for the retrieval of SARS-CoV-2 reservoir studies.

We performed a systematic literature search on SARS-CoV-2 detection studies using the online databases PubMed (www.ncbi.nlm.nih.gov/pubmed), Web of Science (WoS, www.webofknowledge.com) and bioRxiv/medRxiv for the time period January 1^st^ 2020 until June 23^rd^ 2020 (Fig 1).

The following search terms were used in Pubmed and Web of Science to construct the initial dataset of articles: ((SARS-COV2[All Fields] OR ("COVID-19"[Supplementary Concept] OR "COVID-19"[All Fields] OR "covid19"[All Fields])) AND ("2020/01/01"[PDAT]: "2020/12/31"[PDAT]) NOT ("review"[Publication Type] OR "review literature as topic"[MeSH Terms] OR "review"[All Fields]) AND ("loattrfull text"[sb] AND "humans"[MeSH Terms] AND English[lang])) AND (brain OR cerebrum OR cerebellum OR "nervous system" OR neuron* OR spinal OR brainstem OR olfactory OR nasal OR pharyn* OR trachea OR lung OR airway OR bronch* OR heart OR vascular OR arterial OR vena OR "blood vessel" OR "lymph node" OR "lymph vessel" OR lymphatic OR thymus OR spleen OR "bone marrow" OR tonsils OR blood OR oral OR mouth OR? esophagus OR stomach OR pancreas OR liver OR "gall bladder" OR gut OR intestin* OR salivary OR saliva OR stool OR faeces OR kidney OR urethra OR ureter OR bladder OR kidney OR urine OR testit OR epididym* OR prostate OR penis OR sperm OR semen OR ovari* OR uterus OR vagina* OR placenta* OR tegument OR skin OR muscle* OR bone OR joint OR hypothalamus OR pituitary OR thyroid OR adrenal OR reservoir OR autopsy OR cardiovascular OR immunological OR endocrinal OR genital OR urinary OR digestive OR respiratory). In addition, all preprint articles on SARS-CoV-2 were included in the screening strategy.

The total set of 11700 identified articles were pooled in EndNote (version X9.3.3), duplicates removed and a first filtering was performed based on following exclusion criteria: review or editorial articles, non-English manuscripts, non-human studies and no full text available. The remaining set of articles was manually evaluated based on their title, abstract or full text for relevance concerning anatomical compartments, SARS-CoV-2 detection and ACE2 receptor expression. During this process, the snowball method was also used to identify additional papers that were missed by the systematic literature screening approach.

## Results and discussion

### Systematic literature screening

**Fig 2:**
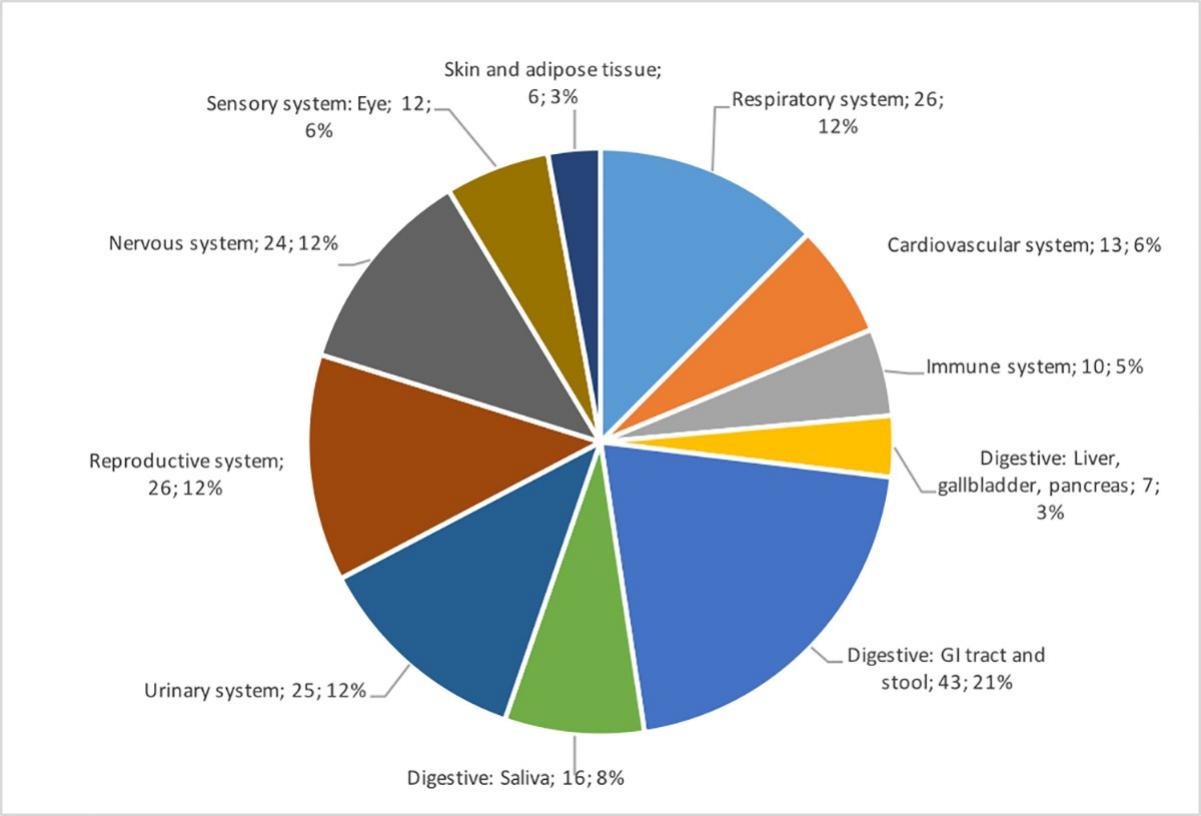
Overview of organ systems studied in SARS-CoV-2 human reservoir studies for the time period January 1^st^ to June 23^rd^ 2020. Next to the widespread use of nasopharyngeal or oropharyngeal swabs (not included in the graph), a full range of SARS-CoV-2 reservoir studies were identified which examined a vast array of human tissues and body fluids.

**Fig 3:**
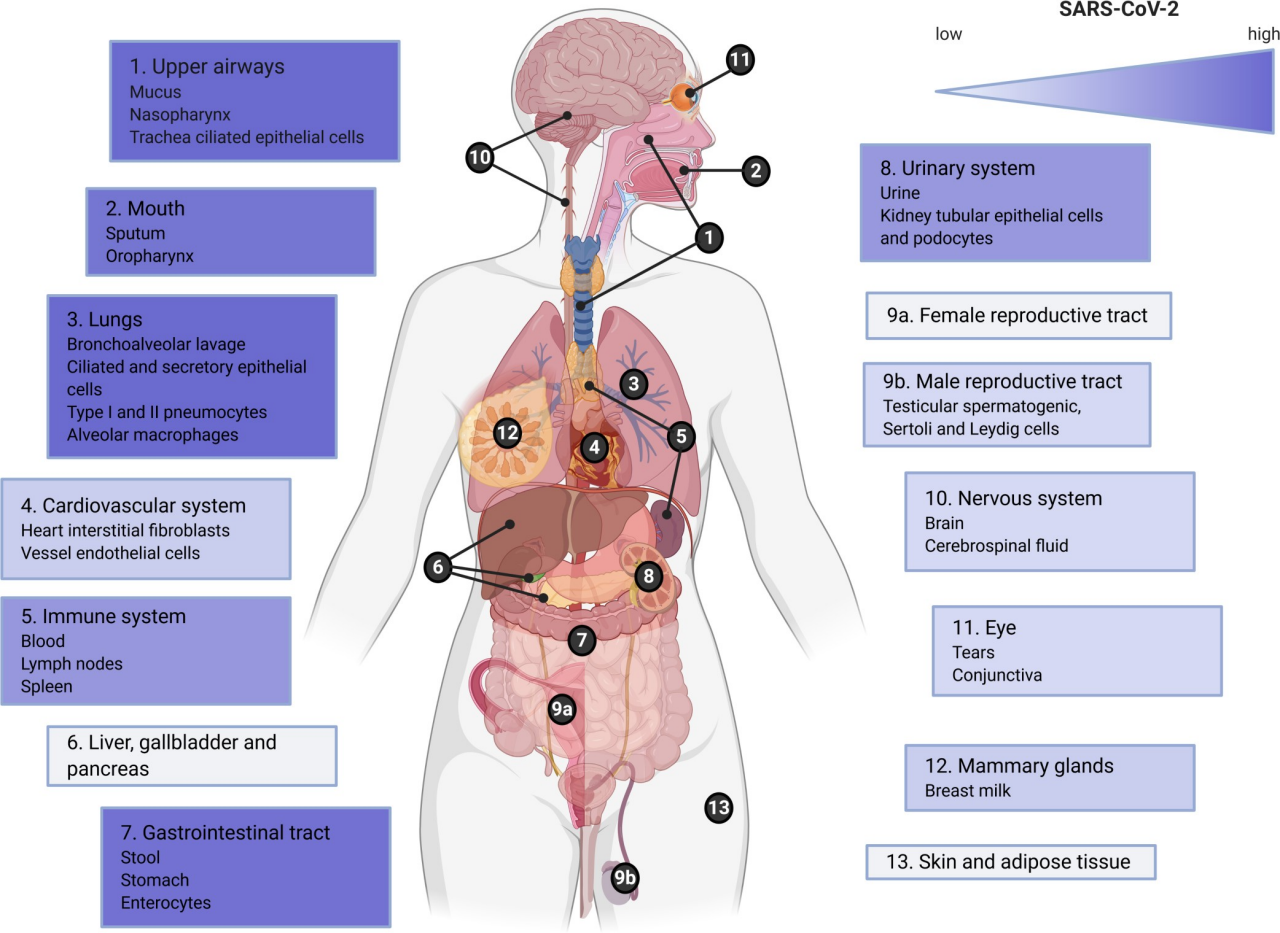
Overview of SARS-CoV-2 presence in the human body. Gradient color (purple) indicates low to high evidence for SARS-CoV-2 detection in this organ, tissue or body fluid. Highest expression was found in upper airways, lungs and oral cavity together with the gastrointestinal tract and urinary system. This figure was created with BioRender.com.

**Fig 4:**
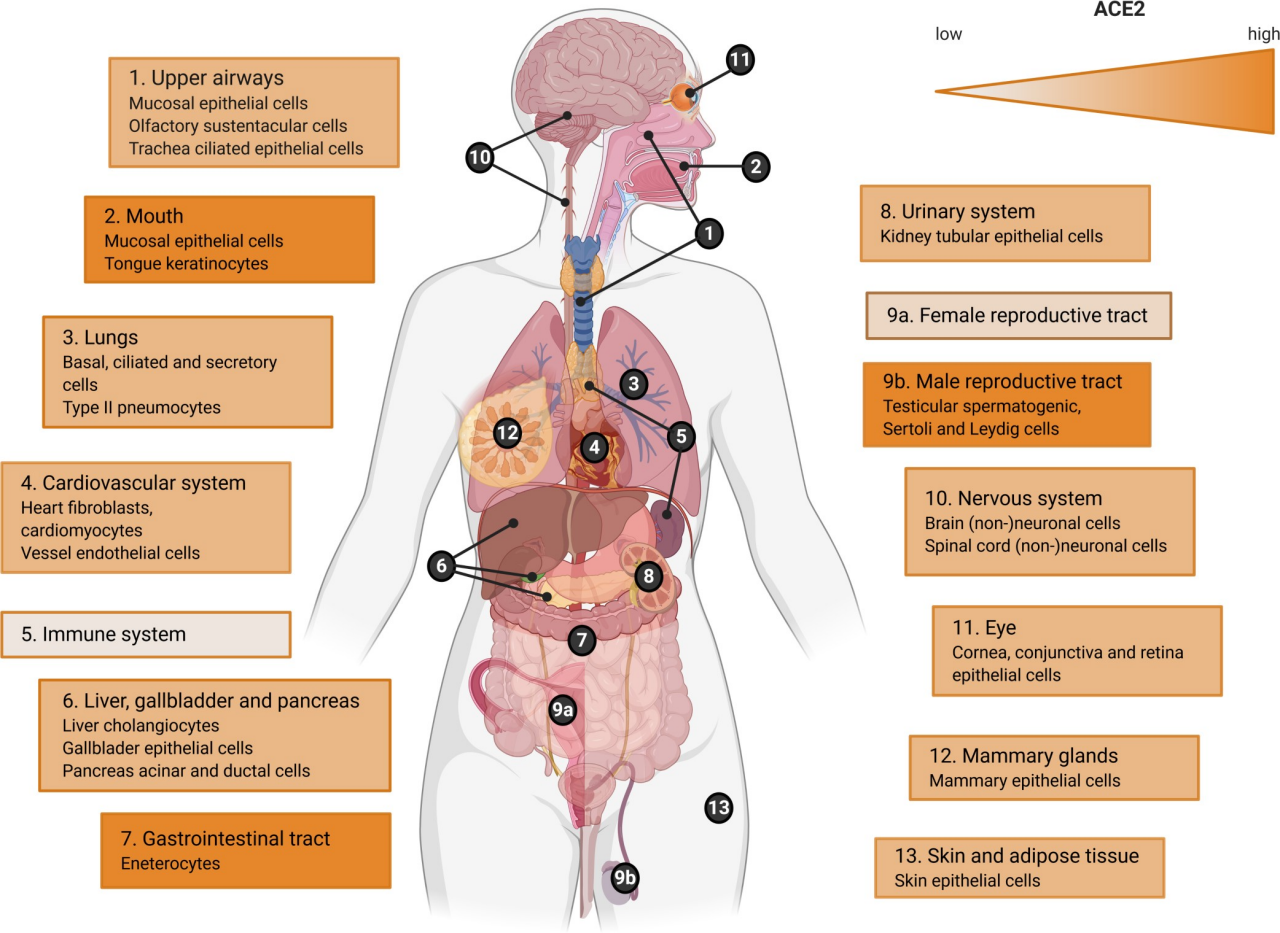
Overview of ACE2 expression levels in the human body. Gradient color (orange) indicates low to high evidence for ACE2 expression in this tissue or body fluid. Highest expression was detected in the oral cavity, gastrointestinal tract and the male reproductive system. This figure was created with BioRender.com.

The screening resulted in 182 articles that were included in this systematic review of which 113 were retrieved from the Pubmed/WoS database and 69 from Bio/MedRxiv (Fig 1)[15–196]. In the examined time period January 1^st^ to June 23^rd^ 2020, a full range of SARS-CoV-2 studies were identified which investigated viral presence in a vast array of human tissues and body fluids spread across the human body (Fig 2). Therefore, information on the presence of SARS-CoV-2 viral fragments or particles and ACE2 receptor expression is organized by organ system in a body map (Figs 3 and 4) and this review will further systematically address major clinical symptoms, detection of SARS-CoV-2 and ACE2 expression levels in each organ system.

Across 182 articles, the detection of SARS-CoV-2 was performed with different methods, with RT-qPCR-based detection of viral RNA and microscopy-based detection (electron and fluorescence microscopy) of viral RNA/protein being the most popular techniques. It is important to note that these techniques do not offer definite proof for the presence of infectious virus, therefore we summarized the level of evidence of viral presence for each organ system (Table 1).

**Table 1:** Overview of SARS-CoV-2 detection in the human body, organized per organ system. Organ systems are shown on the left, followed by type of virus detection and amount of positive samples.

	RT-qPCR (viral RNA)	Electron/Fluorescence Microscopy/Immunohistochemistry (viral RNA or protein)	Culturing virus out of (liquid) biopsies (infectious particles)
**Respiratory system**			
Naso- and/or oropharyngeal swabs	used as diagnostic COVID-19 test*	N.D.	Virus can be isolated out of 17–100% of qRT-PCR-positive samples [34–40]
Sputum	used as diagnostic COVID-19 test*	N.D.	Virus can be isolated out of 30–100% of qRT-PCR-positive samples [35, 36, 38–40]
BAL	used as diagnostic COVID-19 test*	N.D.	N.D.
Trachea	12/12 [19]	tracheal epithelial cells (EM) [19], RNA-ISH [21]	N.D.
Lung	12/12 [19]	type I pneumocytes (EM) [17, 19], RNA-ISH [21]	N.D.
		type II pneumocytes (EM [17], IHC [20], RNA-ISH [21]	N.D.
		alveolar macrophages (EM) [17]	
**Cardiovascular system**			
Heart biopsies	12/15 [19, 42]	interstitial cells of the myocardium [43]	N.D.
Blood vessels	N.D.	blood vessel organoids (FM) [44]	blood vessel organoids can productively be infected [44]
**Immune system**			
Immune cells	RNA seq: rare reads [50, 52]	macrophages [16], virus like particles in CD4+ T cells [53]	N.D.
Blood and plasma	52/384 [52–55]	N.D.	N.D.
Immunesystem-related biopsies	spleen and lymph node [23, 42]	N.D.	N.D.
**Digestive system**			
Stool	504/874 [9, 30, 55, 60–90]	N.D.	two reports isolated infectious virus [77, 79]
Rectal swabs	22/77 [55, 72, 86, 91, 92]	N.D.	N.D.
Saliva	683/758 [89, 94–106]	N.D.	N.D.
Gut biopsies	2 severe patients: esophagus, stomach, duodenum and rectum [9]	gastric, duodenal and rectal epithelia (FM) [79]	N.D.
	large intestines [19]	large intestines lumen (EM) [19]	N.D.
**Urinary system**			
Urine	23/467 [36, 39, 68, 72, 77, 87–89, 92, 132–134, 142]	N.D.	one report isolated infectious virus [135]
Kidney biopsies	3/3 severe patients [19]	tubular epithelium, podocytes and endothelium (EM, IHC) [19, 125–127]	
		kidney organoid (FM) [44]	kidney organoids can productively be infected [44]
**Reproductive system**			
Semen	6/85 [136, 137, 141, 142]	N.D.	N.D.
Prostate secretions	0/23 [143]	N.D.	N.D.
Testis biopsies	0/1 [137]	spermatogenic cells, Sertoli cells and Leydig cells (IHC) [20]	N.D.
Vaginal swabs	0/35 [144]	N.D.	N.D.
Placental swabs, cord swabs and/or amniotic fluid	0/12 [148–150]	N.D.	N.D.
Breast milk	5/42 [156–159]	N.D.	0/9 [159]
**Nervous system**			
Cerebrospinal fluid	4/18 [160, 161, 167–172, 174]	N.D.	N.D.
Brain biopsies	8/34 [19, 173, 174]	N.D.	N.D.
**Sensory system: Eye**			
Tear samples	1/94 [182, 188, 189]	N.D.	N.D.
Conjunctival swabs	8/222 [182–188]	N.D.	N.D.
**Skin and adipose tissue**			
Skin	N.D.	N.D.	N.D.
Adipose	N.D.	N.D.	N.D.

RT-qPCR; reverse transcriptase quantitative polymerase chain reaction, EM; electron microscopy, FM; fluorescence microscopy, IHC; immunohistochemistry, ISH; in situ hybridization, N.D.; not determined. * RT-qPCR data on nasopharyngeal and oropharyngeal swabs, sputum and BAL are not mentioned, as these often function as diagnostic marker for further analyses. Thus, a definite analysis of the exact amount of positive samples is difficult to assess.

### Respiratory system

The first brief reports describing pneumonia due to infection with the new coronavirus were published in the NEJM on January 24^th^ 2020 [15] and Nature on February 3^rd^ 2020 [16]. In these reports, initial identification of SARS-CoV-2 was done via sequencing and phylogenetic analysis on lower respiratory tract and broncho-alveolar lavage (BAL) fluids collected from patients in Wuhan from December 21^st^ 2019 onwards. These patients had been present at the Huanan Seafood market close to the time of symptom onset. They presented with pneumonia of unknown etiology and showed symptoms ranging from common cold, fever, dry cough to dyspnea. In some cases, but mostly in elderly patients, these symptoms progressed to the development of severe acute respiratory syndrome (SARS), similar to the well-known acute respiratory distress syndrome (ARDS) [7]. The pathologies underlying these symptoms usually start with alveolar damage including alveolar edema, vascular decongestion and mild inflammatory infiltration. Later in infection, a diffuse alveolar damage in the organizing phase with reactive type II pneumocyte hyperplasia is observed. Whether or not this will lead to lung fibrosis is not clear yet [17]. In rare cases, excessive hypercoagulation with pulmonary embolisms can lead to sudden death [18].

Presence of SARS-CoV-2 particles has been described in different parts of the respiratory tract, including the nose, pharynx, trachea and lungs. SARS-CoV-2 viral RNA and/or antigens were mainly observed in ciliated respiratory epithelial cells and type I and II pneumocytes, but also in alveolar macrophages [19–21]. In addition, single cell RNA sequencing (scRNA-seq) studies recovered SARS-CoV-2 viral reads from secretory and ciliated epithelial cells in BAL [22] and bronchiolar protected specimen brushes (PSB) [23] from COVID-19 patients. SARS-CoV-2 viral transcripts were also detected in macrophages and neutrophils obtained from BAL, albeit at low levels [22, 23]. Whether this results from direct infection or phagocytosis of infected cells or viral particles remains to be elucidated.

To gain a better understanding of the epithelial subtypes targeted by the virus, Ravindra et al. [24] performed scRNA-seq analysis on *in vitro* infected human bronchial epithelial cells. This study pointed to the ciliated epithelial cells as the major initial target of infection and this was confirmed by EM analysis. At later stages of infection, virus was also detected in other epithelial subsets, including basal, club and BC/club cells. Surprisingly, this study pointed out that ACE2 expression poorly correlated with SARS-CoV-2 detection on a per cell basis. ACE2 was mainly expressed in ciliated cells, club cells and to a lesser extent type II pneumocytes [24, 25]. In this context, ACE2 expression is seemingly higher in the upper airways than in lower airways, while the latter are more affected by SARS-CoV-2 replication [26].

Viral replication in the respiratory tract results in viral shedding in mucus, as evidenced by multiple studies showing viral RNA in nasal and throat swabs. These swabs remain positive for up to 15 days post infection, indicating that viral particles or fragments of viral RNA are still present [27]. Some researchers even demonstrate viral shedding up to 60 days after onset of symptoms [28–30]. Such prolonged viral RNA shedding was mainly observed in elderly patients [31]. Still, relapse involving aggravation of pulmonary dysfunction has only rarely been reported [32]. Moreover, in immunocompromised patients persistent viral shedding and positive PCR with threshold C_t_’s of 30 have been reported [33].

Although viral RNA is routinely detected by RT-qPCR from both upper and lower respiratory tract samples and in particular cases can remain detectable up to 2 months after the onset of symptoms, this does not prove that replication is ongoing in the sample sites and that patients are still infectious. Several studies have isolated infectious virus, that can be propagated *in vitro*, both from upper and lower respiratory tract samples [34–40]. Longitudinal studies indicate that successful viral culture is mostly established from samples obtained within 9 days of onset of symptoms [34, 36, 37]. Interestingly, several studies observed a clear correlation between the viral load as assessed by RT-qPCR and the infectivity of the sample, although the reported threshold C_t_ values that would permit infectivity range from < 24 to < 33 [34, 35, 38], which hampers the use of RT-qPCR as a surrogate diagnostic to predict patient infectivity.

Also, viral loads detected in asymptomatic or minimal symptomatic patients were similar to those of symptomatic patients, hinting towards the transmission potential of asymptomatic patients. This is confirmed in a prevalence study conducted in a nursing home, showing that viral loads were not different between symptomatic, asymptomatic and presymptomatic residents and that viable virus could be isolated from 6 days prior to 9 days past the onset of symptoms [37]. In conclusion, the respiratory tract is the major site of infection, and although virus can be detected for up to 2 months post infection, most studies indicate infectivity is highest in the two weeks post-infection.

### Cardiovascular system

The majority of humans that test positive for SARS-CoV-2 infection do not develop fulminant cardiovascular disease during the acute infection. However, about 20% of the patients admitted to intensive care units develop acute cardiac injury during the course of infection [7, 8]. Whether SARS-CoV-2 facilitates the reported cardiac injuries via direct infection or by triggering inappropriate immune activation is not known. The study of Remmelink et al. [41] reports quantification of viral RNA in the heart in post-mortem patient samples, but did not observe specific viral organ injuries [41]. Two other studies used electron microscopy to detect virus, and of these studies one was able to detect viral RNA, but not viral particles, in the heart [19]. The other study found virus particles in cytopathic, structurally damaged interstitial cells, but not in cardiac myocytes [42]. It is therefore more likely that cardiac injury in patients occurs by inflammation rather than direct infection. Interestingly, one autopsy series of three COVID-19 patients demonstrated endothelitis in vascular beds of different organs and detected viral inclusions via EM in kidney endothelial cells of one patient [43]. Susceptibility of endothelial cells to SARS-CoV-2 infection is furthermore supported by *in vitro* infection studies with blood vessel organoids [44] and pluripotent stem cell-derived cardiomyocytes. In line with this, relatively high expression of ACE2 in heart tissue supports the potential of direct infection [25, 45]. Furthermore, increased expression of ACE2 in patients with heart failure was observed, indicating a potential risk group [46–49]. Although *in vitro* infection studies and expression of ACE2 are suggestive for active infection of the heart, it remains unclear whether this results into cardiac injury as post-mortem studies are inconclusive on the matter. Therefore further research on post-mortem samples or biopsies taken during infection are required to verify if the heart and vasculature could potentially serve as a site of prolonged virus persistence.

### Immune system

Even though immune responses during infection with SARS-CoV-2 can lead to severe complications, no viral replication is observed in immune cells from patients. Interestingly, there are rare reports of viral RNA detection in RNA-seq data from peripheral blood mononuclear cells (PBMCs) [50–52]. In addition, post-mortem studies showed presence of SARS-CoV-2 RNA and antigens in draining lymph nodes and spleen, predominantly in macrophages [19, 20]. One *in vitro* study used virus isolated from patients to test replication kinetics in PBMCs but could not find evidence of SARS-CoV-2 propagation in this heterogeneous population of cells, despite the observation of viral-like particles in primary CD4^+^ T cells by electron microscopy [53]. Detection of viral RNA has been observed in plasma of severe patients, but it remains questionable whether this RNA originates from actual virus particles or merely represents the potentially infectious viral genome [54, 55]. Therefore, detecting RNA in plasma does not directly translate into the presence of infectious particles. In any case, detection of SARS-CoV-2 in multiple organs indicates that infectious virus/RNA is circulating in at least part of the COVID-19 patients. Furthermore, using RNA sequencing data it was found that immune cells barely express the ACE2 receptor required for viral entry nor any other of the major entry proteins [50, 56, 57]. These studies provide the ground for the hypothesis that virus detected in immune cells results from cellular entry by phagocytosis. Since no viral replication was observed thus far in immune cells, it seems that immune cells do not function as a functional reservoir for SARS-CoV-2.

### Digestive system

Gastro-intestinal (GI) tract symptoms are reported in approximately 10–15% of COVID-19 cases and typically include diarrhea and to a lesser extent nausea, vomiting or abdominal pain [9, 58, 59]. Patients with only GI symptoms were more likely to be diagnosed later than patients with additional respiratory complaints and are more likely to be positive for SARS-CoV-2 RNA in fecal samples [60].

Stool samples from 874 patients across 34 studies were examined of which 504 (57.67%) resulted in a positive RT-qPCR for COVID-19 irrespective of disease severity [9, 30, 55, 60–90]. Five studies also included rectal swabs of 77 patients of which 22 tested positive (28.5%) [55, 72, 86, 91, 92]. Stool samples remained positive well after NP swabs returned negative with a range of 1–47 days, indicating longer and extended viral shedding via this route. Also, infectious virus could be isolated from stool samples in at least two reports, indicating the possibility of feco-oral transmission but no reports have shown direct evidence of this occurring [77, 79]. Indeed, stability of SARS-CoV-2 was tested in faeces *in vitro* and was shown to be stable and infectious for several hours [93].

Saliva samples from 758 confirmed COVID-19 patients across fourteen studies yielded high positive SARS-CoV-2 detection results which are often in concordance with matched NP swabs (657/758 patients, 86.7%)[89, 94–106]; however, in a limited set of cases (26 patients) saliva samples were reported positive with a negative NP swab [95, 98, 99, 106]. SARS-CoV-2 could be detected in saliva 10–37 days after onset of symptoms [97, 100, 102] and in two-out-of-three studies was shown to yield higher viral load titers than NP swabs [103, 104, 107]. In addition, SARS-CoV-2 viral particles were shown to be stable in artificial saliva up to 90 minutes [108, 109], further confirming this as a major viral shedding route.

From immunofluorescence and scRNA-seq studies it became apparent that ACE2 expression is high in epithelial cells across the entire gastro-intestinal tract including the oral mucosa of the tongue and enterocytes from the ileum and colon [110–113]. As described above, SARS-CoV-2 has already extensively been recovered from saliva samples and oral swabs. However, whether this results from actual viral replication in the oral (tongue) mucosa and/or salivary glands or is merely a spill-over from the pharynx remains unclear. Nonetheless, the apparent loss of taste in certain COVID-19 patients additionally suggests that SARS-CoV-2 replicates in cells aligning the tongue [114, 115]. Likewise, there is a limited number of studies performed which actually examine viral presence in GI tissues. One study by Lin et al. performed endoscopic sampling in 6 patients of which 2 severe patients tested positive for viral RNA measured by RT-qPCR from the esophagus, stomach, duodenum and rectum [9]. Only 1 out of 4 non-severe cases tested positive in the duodenum. Xiao et al. confirmed these findings by visualizing SARS-CoV-2 viral capsid via intracellular staining of gastric, duodenal, and rectal epithelia in GI tissues [79]. One post-mortem study by Bradley et al. visualized viral particles in the lumen of the large intestines via electron microscopy and found borderline positive RT-qPCR results from tissue biopsies in the large intestines (C_t_ value between 37–40), but not in the small intestine [19]. Finally, Lamers et al. demonstrated that enterocytes of *ex vivo* organoids are infectable with SARS-CoV-2 and that the virus can replicate, indicating that these cells are permissive for SARS-CoV-2 infection [116].

Overall, these findings indicate the broad presence of SARS-CoV-2 RNA or viral fragments in the GI tract with a preference for saliva and stool. Indeed, live virus could be isolated from stool, although from a limited set of patients. Therefore, further studies are needed to map the actual viral presence, especially in gut tissues.

Recurring clinical features of the other solid organs of the digestive tract (liver, gallbladder, pancreas) mainly include liver injury with abnormal liver tests in 35–56% of the COVID-19 patients for aspartate aminotransferase (AST), alanine aminotransferase (ALT) and bilirubin which is probably due to the immune cascade in the body [117, 118]. In addition, there is a single case report indicating a rare onset of SARS-CoV-2-induced pancreatitis in two patients and one cohort study describing pancreatic injury in 10% of COVID-19 patients, indicated by elevated amylase (13/121 patients) or lipase levels (12/121 patients), of which three developed pancreatitis. The limited number of reports indicate that pancreatic injury is often overlooked, hence there is in sufficient data on the widespread presence of this illness [119, 120]. To date, there are no reports on the possible presence of SARS-CoV-2 in these organs, although ACE2 expression can be high and is found in liver cholangiocytes, TROP2+ liver progenitor cells, gall bladder epithelium, pancreatic exocrine glands and islets [49, 121, 122]. Therefore, tissue biopsies with immunohistochemical viral staining or RT-qPCR for viral RNA (i.e. from post-mortem studies) could deliver necessary information, but so far robust data is lacking to conclude that solid organs of the digestive tract play a major role in SARS-CoV-2 infection.

### Urinary system

Routine urinalysis of COVID-19 patients shows abnormalities such as proteinuria, hematuria and leukocyturia in up to 75% of the cases [123, 124]. Further, up to 27% of hospitalized COVID-19 patients even develops acute renal failure, especially elderly with comorbidities such as hypertension and heart failure [10, 125]. Tubule degeneration, necrosis and to a lesser extent renal thrombotic micro-angiopathy typically account for acute renal failure. These observations indicate that the human kidney may be a target for SARS-CoV-2. Indeed, viral RNA, proteins and particles have been found in kidney tubular epithelium, podocytes and to a lesser extent in kidney endothelium of deceased COVID-19 patients [19, 44, 125–127]. This pattern is consistent with ACE2 distribution, as ACE2 is highly expressed onto proximal tubule cells [128–131]. Of note, SARS-CoV-2 was also able to infect human kidney organoids *in vitro* [44]. Despite reports of SARS-CoV-2 presence in the kidney, viral shedding in urine is rather rare, as viral RNA could only be detected in urine samples of 3–4% of COVID-19 patients [36, 39, 68, 72, 77, 87–89, 92, 132–134]. However, one study was able to isolate infectious virus particles out of positive urine samples [135]. Further, viral shedding in urine may continue even after respiratory shedding has ceased, indicating that the human urinary tract may act as a long-term source of SARS-CoV-2 detection[68].

### Reproductive system

Except for few reports on testicular pain, fertility problems or other genital tract-related disorders due to COVID-19 have not been described to date [136]. Nonetheless, SARS-CoV-2 proteins have been found inside spermatogenic cells, supporting Sertoli cells and testosterone-producing Leydig cells of a COVID-19 patient [20]. These virus-positive testes did not show any additional histological abnormalities. In contrast, the testes of another COVID-19 patient did not show any presence of SARS-CoV-2 RNA [137]. Interestingly, the spermatocytes, Sertoli and Leydig cells express a high level of ACE2, compared to other cells in the human body [138–140]. In line with SARS-CoV-2 protein detection in testes, one study detected SARS-CoV-2 RNA in 6 out of 38 semen samples from COVID-19 patients. From the positive samples 4 were collected at the acute stage of infection, whereas 2 were obtained from recovering patients [141]. Still, in four other studies all semen and prostate secretions of 47 and 23, respectively, COVID-19 patients tested negative for SARS-CoV-2 RNA [136, 137, 142, 143]. Presence of SARS-CoV-2 in the female reproductive tract has not been described so far. A number of vaginal swabs of COVID-19 patients also tested negative for viral RNA [144]. Unlike male gonads, female reproductive organs do not express ACE2 at the bulk level [25, 140]. These data indicate the male, rather than the female, genital tract may be susceptible and permissive for SARS-CoV-2 infection. Still, further studies are necessary to confirm this assumption. Although only one out of five studies detected viral RNA in semen, possible sexual transmission of SARS-CoV-2 and the role of the male genital tract in SARS-CoV-2 infection needs further study.

Regarding SARS-CoV-2-positive mothers, despite vascular abnormalities observed in up to 50% of examined placentas, there is no clear evidence of vertical virus transmission to infants [145, 146]. In line with the absence of virus in placenta, amniotic fluid or cord blood, the human placenta does not express high levels of ACE2 [147–151]. In some cases, neonates can still become infected by SARS-CoV-2 after birth through horizontal transmission [152–155]. In addition to respiratory droplets, breastfeeding has been suggested as a mechanism of SARS-CoV-2 transmission, as this has been reported for other RNA viruses, such as HIV. Data are limited, but viral RNA has been identified in breast milk of 5 out of 42 COVID-19-positive breastfeeding mothers so far [156–159]. Although still anecdotal, the most convincing evidence for the possible occurrence of SARS-CoV-2 in milk is provided by a study of milk samples from two SARS-CoV-2-positive breastfeeding mothers, demonstrating SARS-CoV-2 RNA in milk samples from one mother for four consecutive days [157]. Still, it was impossible to recover infectious virus from RNA-positive milk samples, suggesting that breastmilk itself is unlikely to pass on SARS-CoV-2 to infants [159]. However, more evidence is needed to conclude on the definite role of breastfeeding in SARS-CoV-2 transmission.

### Nervous system

The majority of COVID-19 patients (up to 78%) show neurological symptoms ranging from headache, loss of smell (anosmia) and taste (ageusia), imbalance, impaired consciousness, delirium and paresthesia to extremity paralysis and convulsions [11, 114, 115, 160–163]. Severe neurological symptoms can mostly be accounted to abnormalities located in the brain(stem) and spine such as edema, hemorrhages and thrombotic events with or without stroke, demyelination and encephalomyelitis [163–166]. This has urged many researchers to look closer into the nervous system of COVID-19 patients. In COVID-19 cases with severe neurological symptoms, viral RNA has been identified in 4 out of 8 cerebrospinal fluid (CSF) samples [160, 161, 167–172]. Moreover, one autopsy study found traces of viral RNA via RT-qPCR in the brain of 8 out of 22 deceased COVID-19 patients [173]. Yet, two other studies failed to identify virus particles or RNA in brain autopsies and CSF analysis of 12 and 10, respectively, deceased COVID-19 patients [19, 174]. Therefore, it remains unclear whether severe neurological manifestations are triggered by direct viral-induced damage or virus-induced endothelial and/or cytokine disturbances. Nonetheless, ACE2 is expressed in both neuronal and non-neuronal cell types in the human central nervous system, especially in the spinal cord, dorsal root gangla, brainstem substantia nigra, choroid plexus, hypothalamus, hippocampus, middle temporal gyrus and posterior cingulate cortex [175, 176]. This hints towards the possibility of SARS-CoV-2 invasion of the central nervous system. In contrast to severe neurological symptoms, ageusia and anosmia are often devoid of any obvious lesions [114, 115]. Direct viral damage to the olfactory and gustatory receptors or neurons has been proposed as mechanism of sensorineural olfactory loss, but the absence or presence of SARS-CoV-2 inside olfactory epithelial or neuronal cells has not been described so far. This is probably related to technical difficulties during COVID-19 patient autopsy, such as inaccessibility of the olfactory bulb and epithelium without the use of electric saws to cut into the skull. This generates potentially virus-loaded aerosols, necessitating the use of additional, sometimes difficult to take, precautions. Interestingly, non-neuronal cells rather than neuronal cells residing in the olfactory epithelium and bulb express ACE2 and TMPRSS2 [175, 177–179]. This indicates that SARS-CoV-2 replication in the olfactory epithelium might induce anosmia through perturbation of supporting cells, rather than direct neuronal infection. Together, these findings indicate that SARS-CoV-2 is able to reach the nervous system.

### Sensory system: Eye

Ocular symptoms remain rare in COVID-19 patients but reported symptoms in hospitalized patients include: dry eyes, blurred vision, foreign body sensation and conjunctivitis with conjunctival congestion. However, there remains a debate whether this is directly caused by the virus [180, 181]. As SARS-CoV was readily detectable in tears, Xia et al. investigated the viral presence of SARS-CoV-2 RNA in tears and conjunctival swabs from 30 COVID-19 patients who were sampled twice longitudinally [182]. Only one patient showed a positive PCR test on tears and ocular surface swabs with onset of conjunctivitis and conjunctival congestion. However, the virus could not be isolated or cultured *in vitro*. This was also reported by Xie et al., Kumar et al., Wu et al., and Zhou et al. who detected SARS-CoV-2 RNA in ocular surface swabs or conjunctival secretions of 2/33, 1/45, 2/28 and 1/63 patients, respectively [183–186]. One case report performed longitudinal sampling in a single patient and confirmed a positive signal of conjunctival swabs up to 17 days after onset of disease [187]. In contrast, Seah et al. and Xu et al. could not replicate these findings and did not detect SARS-CoV-2 in 64 tear samples from 34 patients [188] or in ocular surface swabs from 22 patients [189]. Reports on ACE2 expression find a limited expression at the retina and specifically in the retinal epithelium [180]. Moreover, ACE2 and TMPRSS2 are co-expressed in corneal and conjunctival epithelial cells according to scRNA-seq and immunohistochemical analyses, identifying the ocular surface as a potential viral entry site [190, 191]. Therefore, infection could occur via droplets entering the eye and travel via the nasolacrimal canal to the respiratory tract.

In total across eight studies examining ocular presence of SARS-CoV-2 RNA, 1/94 patients tested positive for tear or conjunctival secretion samples and 8/222 ocular surface swabs. These low numbers can be influenced by the time of sampling and by the onset of conjunctivitis but show that ocular fluids can contain SARS-CoV-2 viral fragments RNA. However, no reports state that SARS-CoV-2 virus could be isolated from ocular fluids at present.

### Skin and adipose tissue

The documentation and available papers surrounding SARS-CoV-2 in relation to adipose and skin tissue is very limited. About 20% of patients present or develop cutaneous manifestations at or during the onset of COVID-19, but not related to severity of infection. These patients presented themselves with erythema, and a positive diagnostic test for SARS-CoV-2 [192–194]. No tests on skin tissue have thus far been performed, however, and therefore it cannot be excluded as an underlying illness or as a manifestation of the immune response. Remarkably, ACE2 receptor is expressed in skin biopsies, and patients with rash and skin lesions have increased expression of TMPRSS2 (a co-receptor of SARS-CoV-2] [57]. Therefore, rash could also be the symptom of infection leading to lesions, but this needs to be confirmed and further investigated. In adipose tissue thus far no virus has been detected. However, the paper of van der Poort et al. (2020) found that patients that have an increased Body Mass Index (BMI) also have increased leptin levels in serum, which they hypothesize could correlate with severity of the infection [195, 196]. Interestingly, increased expression of the ACE2 receptor in the lung epithelia are positively correlated with obesity, indicating that obese individuals might be at higher risk for SARS-CoV-2 infection [195, 196]. In conclusion, there is no robust evidence to either demonstrate or exclude that skin and adipose tissue harbor SARS-CoV-2 viral particles.

## Conclusion

### SARS-CoV-2 affects many organs throughout the human body

The first studies on SARS-CoV-2 tropism and pathogenesis focused on the lungs, as these were “the viral ground zero”. However, it quickly became clear that SARS-CoV-2 also attacks other organ systems, either by direct viral infection or through indirect effects of the immune response. Our systematic review showed that traces of the virus have been found in multiple organs throughout the body, including the pharynx, trachea, lungs, blood, heart, vessels, intestines, male genitals, brain and kidneys. In line with this, SARS-CoV-2 components were also detected in a variety of body fluids such as mucus, saliva, urine, semen, faeces, cerebrospinal fluid and breast milk. Of note, although SARS-CoV-2 RNA has been detected in plasma of patients with severe disease [54, 55, 197], no study has as yet demonstrated the presence of infectious virus in blood. It therefore remains to be demonstrated that SARS-CoV-2 might infect organ systems by gaining access to the bloodstream. Although highly speculative at the moment, SARS-CoV-2 might cause a persistent chronic infection in certain individuals. In these patients, certain sites might act as a “viral reservoir”, in which the virus can persist for prolonged periods accompanied by recurrent viral shedding. This phenomenon is not new for RNA viruses, as this has already been described in Ebola virus survivors [198]. SARS-CoV-2 persistence and recurrent shedding has already been demonstrated in the respiratory tract [33], but also sites of attenuated immunity such as the testis, eye and brain might potentially preserve the virus for longer times. Nonetheless, this hypothesis should be treated with caution, as the number of studies showing actual infectious virus particles in different organs remains limited so far.

### ACE2 expression and SARS-CoV-2 infectivity: not a perfect match

Given the role of ACE2 as the main cellular entry receptor for SARS-CoV-2 *in vivo*, ample studies have attempted to map ACE2 expression to obtain insight into tissues or cell types that are in theory susceptible to SARS-CoV-2 infection. Interestingly, presence of SARS-CoV-2 components in different tissues does not always correlate with steady-state ACE2 expression levels. For instance, high viral loads are retrieved from the lungs, which generally show rather low ACE2 expression levels. In the gastrointestinal tract, viral loads peak in the colon, while ACE2 expression is higher in the small intestine. Thus, it seems that there is not always a perfect match between ACE2 expression and SARS-CoV-2 detection. A plausible explanation for this apparent mismatch might be the fact that few ACE2 molecules might suffice to cause a productive SARS-CoV-2 infection. Alternatively, cell type heterogeneity in ACE2 expression within a given tissue can also contribute as well as a discordance between ACE2 mRNA expression and cell-surface ACE2 expression. Further in depth analysis of cell-surface ACE2 protein expression is however required to confirm these hypotheses. Of note, most evidence on ACE2 expression in different tissues is based on a steady-state situation in healthy individuals. In diseased individuals, expression levels of ACE2 might differ. Indeed, several comorbidities associated with severity of COVID-19 disease such as smoking, diabetes, COPD, obesity and hypertension are characterized by elevated levels of ACE2 expression in the respiratory tract [199–201]. However, whether severe progression of COVID-19 in these patients is a direct consequence of abundant ACE2 expression and thus increased susceptibility to SARS-CoV-2, or is merely the result from underlying health issues in these individuals (e.g. immunosuppression) is unclear. Counterintuitively, ACE2 receptor abundance in plasma is high in children, who often show minor symptoms upon SARS-CoV-2 infection, while it goes down in the elderly, who are at greater risk of severe illness [202, 203]. This apparent paradox might be explained by the discrepancy between membrane-bound and soluble ACE2 molecules. While the membrane-bound form acts as host cell receptor for SARS-CoV-2, soluble ACE2 may neutralize free virions by shielding the viral binding protein spike (S) [204]. In this way, elevated soluble ACE2 levels in children may help them to contain the virus. Thus, it is clear that researchers only discovered the “tip of the iceberg” in SARS-CoV-2 pathogenesis so far.

### Limitations

As described in this review, only a limited set of studies succeeded in isolating and culturing SARS-CoV-2 viral particles out of a biopsy that were also capable of reinfecting target cells *in vitro*, providing definite proof of replication-competent viral presence of SARS-CoV-2. Importantly, this “golden standard”evidence is currently only available for respiratory and gastro-intestinal tract samples and it remains to be established whether the “viral signal” detected in other organs systems via RT-qPCR or microscopy derives from genuine infective viral particles [205]. For instance, in electron microscopy, viral particles might be mistakenly identified over other endogenous vesicles or endocytic bodies [206] and a (borderline) positive signal in RT-qPCR could be due to leftover viral RNA fragments rather than replication-competent virus. Indeed, as virus is phagocytosed by immune cells and these cells can travel throughout the body and invade different tissues, weak signals detected via RT-qPCR might derive from immune cells that phagocytosed virus at distant locations.

An important limitation of this study is that information on viral load/ACE2 expression in patient samples is biased towards sample types that are easily accessible such as body fluids or blood cells. For instance, although scRNAseq studies could yield interesting information on SARS-CoV-2-targeted cell types, the epithelial cells, which constitute the major target of SARS-CoV-2 according to the available evidence, are underrepresented in respiratory samples obtained from patients. Most information on less accessible tissues/cell types therefore derives from autopsy studies, which are biased towards analyses of the subset of patients with critical COVID-19 disease and also represents an endpoint analysis. These limitations could be addressed with an appropriate animal model, replicating the pathogenesis of human COVID-19 [207]. However, although the mild COVID-19 phenotype and viral replication in the respiratory tract can be mimicked in non-human primates, hamsters, ferrets and cats, none of these animals features the cytokine storm and coagulopathy that characterizes severe COVID-19 in humans [208]. Therefore, this review primarily focused on SARS-CoV-2 studies in the context of humans.

Finally, difficulties inherent to the heterogeneous SARS-CoV-2 research are the variability in study design, patient numbers and characteristics, sampling timing and testing procedures. Furthermore, the included preprint articles should be treated with caution because no strict peer review process was performed. This systematic review focused on research articles available in English and on studies reporting human data.

These limitations should be considered while interpreting these data.
